# Decoding diabetic kidney disease: a comprehensive review of interconnected pathways, molecular mediators, and therapeutic insights

**DOI:** 10.1186/s13098-025-01726-4

**Published:** 2025-06-04

**Authors:** Esienanwan Esien Efiong, Kathrin Maedler, Emmanuel Effa, Uchechukwu Levi Osuagwu, Esther Peters, Joshua Onyeka Ikebiuro, Chisom Soremekun, Ugwunna Ihediwa, Jiefei Niu, Markéta Fuchs, Homa Bazireh, Akang Leonard Bassey, Peter Uchenna Amadi, Qiuling Dong, Njogu Mark Kimani, Rebecca Chinyelu Chukwuanukwu, Emmy Tuenter, Sapna Sharma, Harald Grallert

**Affiliations:** 1 Research Unit of Molecular Epidemiology, Institute of Epidemiology, Helmholtz Zentrum München, 85764 Neuherberg, Germany; 2 Department of Biochemistry, Faculty of Science, Federal University of Lafia, PMB 146, Lafia, 950101 Nigeria; 3 Islet Biology Laboratory, Centre for Biomolecular Interactions, University of Bremen, Bremen, Germany; 4 Division of Nephrology, Department of Internal Medicine, Faculty of Clinical Sciences, University of Calabar, PMB 1115, Calabar, 540271 Nigeria; 5 School of Medicine, Bathurst Rural Clinical School, Western Sydney University, Bathurst, NSW 2795 Australia; 6 Faculty of Science, Masaryk University, 60200 Brno, Czech Republic; 7 Human and Animal Physiology Group, Wageningen University and Research, Wageningen, The Netherlands; 8 Department of Immunology and Molecular Biology, School of Biomedical Sciences, Makerere University College of Health Sciences, Kampala, Uganda; 9 Emergency Medicine, Royal Cornwall Hospital, Truro, TR1 3LJ UK; 10 Faculty of Medicine, Ludwig-Maximilians-University Munchen, 81377 Munich, Germany; 11 Developmental Biology and Cancer Research & Teaching Department, UCL Great Ormond Street Institute of Child Health, 30 Guilford Street, London, WC1N 1EH UK; 12 Department of Pediatrics, Faculty of Medicine and Dentistry, University of Alberta, Edmonton, AB T6G 2R3 Canada; 13 Department of Biochemistry, Imo State University, Owerri, 460222 Nigeria; 14 Department of Physical Sciences, University of Embu, P. O. Box 6, Embu, 60100 Kenya; 15 Institut Für Organische Und Analytische Chemie, Universität Bremen, Leobener Straße NW2C, 28359 Bremen, Germany; 16 Department of Internal Medicine 3, Uniklinikum Erlangen and Deutsches Zentrum Für Immuntherapie (DZI), Friedrich-Alexander-Universität Erlangen-Nürnberg (FAU), Erlangen, Germany; 17 Immunology Department, Faculty of Medical Laboratory Science Department, Nnamdi Azikiwe University, Awka, Nigeria; 18 Faculty of Pharmaceutical, Biomedical and Veterinary Sciences, Department of Pharmaceutical Sciences, University of Antwerpen, Campus Drie Eiken, Universiteitsplein 1, 2610 Antwerp, Belgium; 19 German Research Center for Environmental Health, Helmholtz Zentrum Munchen, 85764 Neuherberg, Germany

**Keywords:** Diabetic nephropathy, Signal pathways, Chronic kidney disease, End-stage kidney disease, Renin–angiotensin–aldosterone system, Toll-like receptors, Janus kinase/signal transducer and activator of transcription, Nuclear factor-kappa B, Transforming growth factor-beta, Hippo signalling

## Abstract

**Background:**

Diabetic kidney disease (DKD) is a chronic kidney condition that arises from prolonged hyperglycaemia that can progress to kidney failure, severe morbidity, and mortality if left untreated. It is the major cause of chronic kidney disease among people who have diabetes, accounting for a significant percentage of patients with end-stage kidney disease who require kidney replacement therapy.

**Main body:**

In DKD, numerous dysbalanced metabolic, haemodynamic, inflammatory signalling pathways, and molecular mediators interconnect, creating a feedback loop that promotes general kidney damage. Hyperglycaemia is the primary trigger for DKD and leads gradually to oxidative stress, inflammation, extracellular matrix deposition and fibrosis, glomerular hypertension, and intrarenal hypoxia. Key interconnected metabolic pathways are the hyperglycaemia-mediated polyol, hexosamine, protein kinase C, and advanced glycation end-products pathway hyperactivity. Concurrently, hyperglycaemia-induced renin–angiotensin–aldosterone system stimulation, alters the kidney intraglomerular haemodynamic leading to inflammation through Toll-like receptors, Janus kinase/signal transducer and activator of transcription, and nuclear factor-kappa B, transforming growth factor-beta-mediated excessive extracellular matrix accumulation and fibrosis. The resulting death signals trigger apoptosis and autophagy through Hippo, Notch, and Wnt/β-catenin pathway activation and microRNA dysregulation. These signals synergistically remodel the kidneys culminating in intrarenal hypoxia, podocyte dysfunction, hyperfiltration, epithelial-mesenchymal transition, and loss of kidney function. The resulting renal failure further upregulates these death pathways and mediators, giving rise to a vicious cycle that further worsens DKD.

**Conclusion:**

This review provides an overview of the primary molecular mediators and signalling pathways leading to DKD; their interconnectivity at the onset and during DKD progression, the central role of transforming growth factor-beta via different pathways, the Hippo pathway kidney-specific response to hyperglycaemia, and how all mediators and transduction signals result in a vicious circle that exacerbates renal failure. The review gives therapeutic sights to these pathways as druggable targets for DKD management. Understanding these molecular events underlying the pathogenesis of DKD can bridge basic research and clinical application, facilitating the development of innovative management strategies.

## Introduction

Diabetic kidney disease (DKD) is a common and severe microvascular complication of diabetes and is the primary cause of chronic kidney disease (CKD). Despite optimal glycaemic control, DKD remains a major contributor to end-stage kidney disease (ESKD), placing significant pressure on healthcare systems worldwide [1]. The disease accounts for 30 to 40% of patients with ESKD who require kidney replacement therapy [2]. People with DKD had a faster decline of kidney function (50% estimated glomerular filtration rate decline and initiation of kidney replacement therapy) than those with kidney disease without diabetes [3, 4].

An expansion of kidney replacement therapy to meet the rising global demand is untenable, given the high costs and ageing population worldwide. Existing medical treatment does not reliably halt the progression of DKD across the patient spectrum [5]. So, the emerging potential and prospect of personalised care necessitate an in-depth understanding of the heterogeneity of pathophysiologic mediators and therapeutic responsiveness observed in clinical practice [6].

Chronic hyperglycaemia affects all kidney cells, including podocytes, tubular interstitial, endothelial, and mesangial cells (MC), resulting in functional and structural alterations [7]. This triggers morphological changes that involve capillary loss, MC proliferation, extracellular matrix (ECM) buildup, early glomerular hypertrophy, glomerular basement membrane (GBM) thickening, and injury to podocytes and glomerular cells [8, 9]. These abnormalities occur almost simultaneously [7] with the progression to ESKD, which is clinically characterised by glomerular hyperfiltration, increased albuminuria, and a decrease in glomerular filtration rate (GFR) [10, 11].

DKD is a complex disease with numerous interconnected metabolic, pro-inflammatory, and pro-apoptotic pathways that impact haemodynamic abnormalities, glomerular hypertension, and metabolic disorders [12], resulting in a deleterious feedback loop and vicious cycle. These causal mechanisms are interwoven and influence gene regulation and transcription factor activation, both of which have a negative impact on molecular pathways [13].

Hyperglycaemia influences these pathways in complex ways. They include hyperactivity of the renin-polyol and hexosamine paths, protein kinase C (PKC), and formation of advanced glycation end-products (AGEs). This metabolic dysregulation potentiates inflammation by activating Toll-like receptors (TLRs), Janus kinase/signal transducer and activator of transcription (JAK/STAT) signals, and the nuclear factor-kappa B (NF-κB) pathway. These pathways activate transforming growth factor-beta (TGF-β), which mediates fibrosis, extracellular matrix remodelling, apoptosis, and autophagy dysregulation via Hippo, Notch, Wnt/β-catenin activation, and microRNA dysregulation [12–16]. Altogether, these mediators promote intrarenal hypoxia, podocyte dysfunction, hyperfiltration, epithelial-to-mesenchymal transition (EMT), and finally renal failure (Table 1).

**Table 1:** Key signalling pathways and mediators involved in fibrosis and kidney damage in diabetes

Signals	Pathways	Mediators	Effect on fibrosis and inflammation
Hyperglycaemia	RAAS	Ang II, ET-1, TGF-β	Promotes fibrosis via oxidative stress, ECM production
Metabolic Dysregulation	Polyol Pathway	Aldose reductase, Sorbitol	Increases ROS, inflammation, fibrosis
	Hexosamine Pathway	GFAT, UDP-GlcNAc	Leads to ECM overproduction, stress signaling
	AGEs	RAGE, ROS, NF-κB	Promotes inflammation, oxidative stress, fibrosis
	AMPK	AMPK, SIRT1, mTOR	Energy homeostasis, anti-fibrotic effects
Inflammation	PI3K/AKT	PI3K, AKT, mTOR	Regulates cell survival, inflammation, fibrosis
	TLRs	NF-κB, JAK/STAT, IL-1β, TNF-α	Activates immune response, cytokine release, chronic inflammation
	NF-κB	TNF-α, IL-1β, IκB kinase	Promotes inflammation, fibroblast activation, ECM accumulation
	JAK/STAT	STAT3, IL-6, IFN-γ	Cytokine signalling, myofibroblast proliferation
Pro-fibrotic	TGF-β/Smad	Smad2/3, CTGF, α-SMA	Myofibroblast activation, ECM deposition
	Wnt/β-catenin	β-catenin	Drives fibroblast proliferation, EMT, ECM deposition
	Notch Signaling	Notch1-4, Hes1, Jagged	Fibroblast activation, EMT, tubulointerstitial fibrosis
	Hippo	YAP/TAZ, TEAD	Myofibroblast proliferation, ECM production
Hypoxia & Fibrosis Feedback	Intrarenal Hypoxia	HIF-1α, VEGF	Promotes fibrosis through inflammation and oxidative stress

Toll-like receptors (TLRs), Janus kinase/signal transducer and activator of transcription (JAK/-STAT), and nuclear factor-kappa B (NF-κB), transforming growth factor-beta (TGF-β), interleukin-1 beta (IL-1β), tumour necrosis factor alpha (TNF-α), inhibitory kappa B protein (IκB), extracellular matrix (ECM), interleukin 6 (IL-6), interferon-γ (IFN-γ), Suppressor of Mothers Against Decapentaplegic (Smad), connective tissue growth factor (CTGF), renin–angiotensin–aldosterone system (RAAS), angiotensin II (Ang II), endothelin-1 (ET-1), yes-associated protein (YAP), transcriptional coactivator with PDZ binding motif (TAZ), TEA domain (TEAD), reactive oxygen species (ROS), glutamine: fructose-6-phosphate-amidotransferase (GFAT), advances glycaetion end-products (AGEs), receptor for advance glycation end products (RAGE), adenosine monophosphate-activated protein kinase (AMPK), sirtuin-1 (Sirt1), mammalian target of rapamycin (mTOR), phosphoinositide-3-kinase (PI3K), protein kinase B (AKT), hypoxia-inducible factor-1α (HIF-1α), vascular endothelial growth factor (VEGF)

Key pathways of DKD offer numerous druggable targets for future therapeutic intervention aimed at slowing disease progression and preventing or delaying kidney failure. This review is aimed at examining the vicious cycle of interconnected signalling pathways and mediators at different phases of the disease and the central role of TGF-β as an upstream and downstream mediator of DKD via multiple pathways. The goal is to improve tailored patient care and outcomes.

## The activation of deleterious glucose metabolism in DKD

### Polyol pathway hyperactivity

The polyol pathway diverts excess glucose metabolism, leading to sorbitol and fructose accumulation. This raises oxidative stress (OS) and weakens antioxidant defences, contributing to DKD. This process entails two enzymes, aldose reductase and sorbitol dehydrogenase that reduce intracellular nicotinamide adenine dinucleotide phosphate hydrogenase (NADPH) and increase reactive oxygen species (ROS) production [17].

Urinary excretion of sorbitol reflects the degree of polyol pathway activation [17]. DKD activates glucose-dependent cellular stress mechanisms, such as the polyol pathway [18]. This pathway plays a crucial role in the development of DKD by generating osmotic stress and hyperglycaemic OS in renal tissue. Under homeostatic conditions, cellular glucose is oxidised mainly into glucose-6-phosphate, which enters glycolysis to produce energy in the form of adenosine triphosphate [19]. Aldose reductase converts excess glucose to sorbitol, which is slowly metabolised by sorbitol dehydrogenase. Thus, sorbitol accumulation and its poor permeability across cellular membranes cause hyperosmotic stress within cells. This is the primary determinant for the development of cataracts and microvascular complications of diabetes (nephropathy, retinopathy, and neuropathy) [20].

**Fig. 1:**
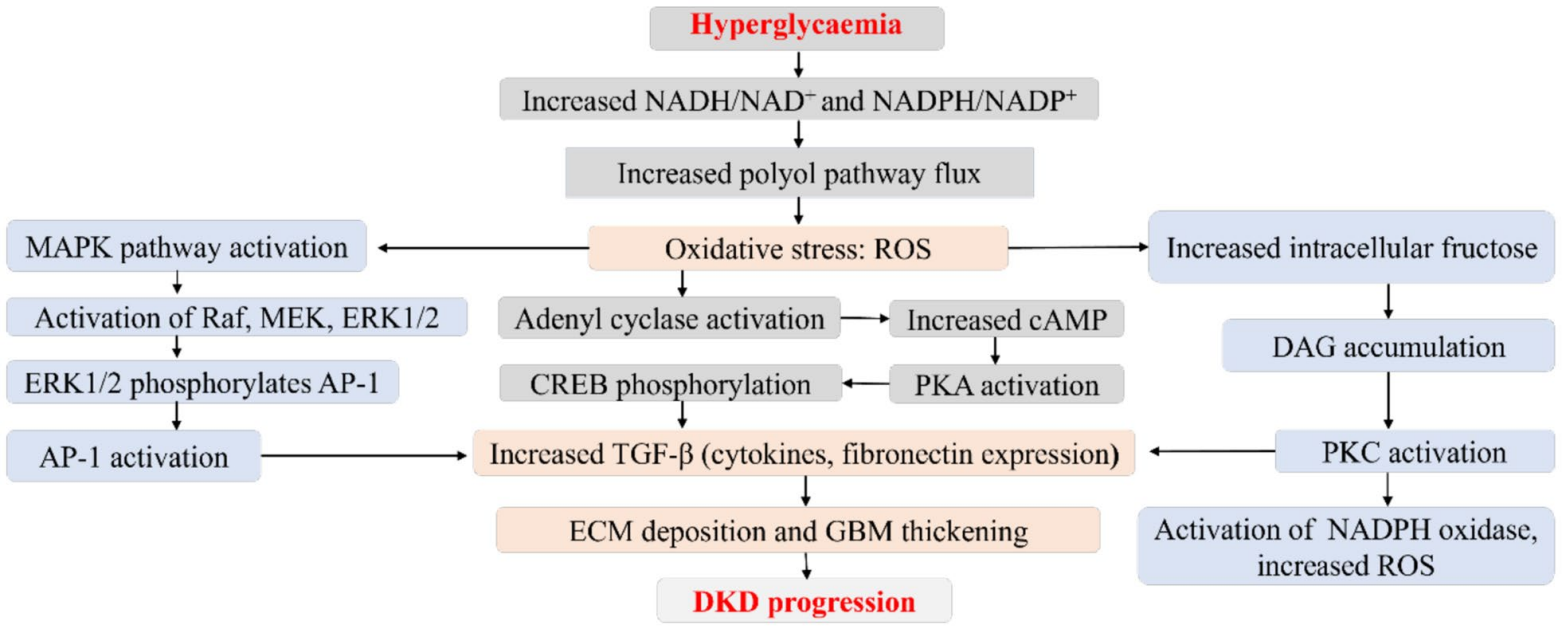
Contribution of chronic hyperglycaemia to increased polyol pathway flux and diabetic kidney disease progression. Increased polyol pathway nux increases oxidative stress, activates the mitogcn-activatcd protein kinase (MAPK), rapidly accelerates fibrosareoma (Raf), mitogcn­activated protein kinase (MEK), extracellular signal-regulated kinases I and 2 (ERKl/2), and transcription factors activator protein-I (AP-I). Increased oxidative stress increases intracellular fructose, causes diacylglycerol (DAG) accumulation, protein kinase C (PKC)/nicotinamide adenine dinuclcotidc phosphate (NADPH) oxidasc activation, and increased reactive oxygen species (ROS). ROS further activates adcnyl cyclase through a series of reactions that culminates in increased transforming growth factor-beta (TGF-β). AP-I and PKC activation also results in high expression of TGF-β. All of which results in extracellular matrix (ECM) accumulation. glomcrular basement membrane (GHM) thickening. and ultimately DKD development

Elevated polyol pathway flux activates the mitogen-activated protein kinase (MAPK), transcription factors activator protein-1 (AP-1), PKC, and cyclic adenosine monophosphate response element binding protein signalling pathways. This results in the upregulation of molecules such as TGF-β, other cytokines, and fibronectin that are associated with the thickening of GBM and ECM deposition in the mesangium [20, 21] (Fig. 1). The inactivation of these mediators results in improved kidney function. For instance, [22, 23] showed that by inhibiting the activity of PKCβ, the β isoform of PKC resulted in improved glomerular endothelial function in insulin-resistant and diabetic conditions.

### Hexosamine pathway, AGEs, and the renin–angiotensin–aldosterone system (RAAS) hyperactivity

Hyperglycaemia induces aberrant hexosamine pathway fluxes, which contribute to diabetes complications by increasing the fructose-6-phosphate concentration and directing it to the hexosamine biosynthetic pathway [12, 24]. Under physiological conditions, hexokinase converts glucose to glucose-6-phosphate, which is then converted to fructose-6-phosphate by phosphoglucoisomerase. Phosphofructokinase catalyses the conversion of fructose-6-phosphate to fructose-1,6-bisphosphate. In hyperglycaemia, however, most of the fructose-6-phosphate is converted into glucosamine-6-phosphate via the enzymatic activity of fructose-6-phosphate aminotransferase, which is then metabolised into various aminohexose derivatives, such as uridine diphosphate N-acetylglucosamine, a precursor for glycosaminoglycans, proteoglycans, glycoproteins, and other amino sugars [7]. The overexpression of fructose-6-phosphate aminotransferase leads to increased gene transcription of fibronectin, plasminogen activator inhibitor-1, and TGF-β in glomerular MCs, leading to expansion and thickness of the GBM [18]. The end-products of the hexosamine pathway phosphorylate transcription factors at serine and threonine residues, leading to overexpression of TGF-β involved in DKD [18, 25].

Long-term exposure to a hyperglycaemic condition increases the risk of AGEs and glycosylation in the kidneys. Prolonged circulating AGEs interact with their receptor, RAGE, causing OS and activating several pathways, including p38, Ras-mediated extracellular signal-regulated kinase (ERK)1/2, stress-activated PKC-Jun N-terminal kinase, MAPK/ERK, and JAK/STAT. This interaction induces endoplasmic reticulum stress, inflammation, and fibrosis, all of which accelerate renal pathology [7]. The synthesis of AGEs is irreversible, and the pathways they stimulate lead to sustained activation of transcription factors like hypoxia-inducible factor-1α (HIF-1α), AP-1, NF-κB, and STAT3 [12]. These cellular perturbations trigger a chain reaction of proinflammatory cytokines, including interleukin-6 (IL-6) and tumour necrosis factor alpha (TNF-α) [5], and activate several overlapping fibrotic pathways [16].

AGEs activate the RAAS, leading to increased glomerular filtration pressure and TGF-β expression [26] which accelerates the course of DKD [27, 28]. The RAAS alters kidney haemodynamics by raising OS and activating proinflammatory pathways. These processes result in glomerular enlargement, which indicates initiation of the profibrotic process at the onset of DKD [12].

### Adenosine monophosphate (AMP)-activated protein kinase (AMPK) hyperactivity

The AMPK signalling pathway is a unique therapeutic target due to its ability to reprogram metabolism at both cellular and systemic levels [29]. AMPK is a cellular energy sensor [30] with aberrant expression in many diseases, such as diabetes, cardiovascular disease, and certain cancers [24]. AMPK mediates the harmonization of various anabolic processes, and its signalling regulation is vital for modulating cell homeostasis [31]. A study in an insulin-resistant animal model found that AMPK activation enhanced lipid and glucose homeostasis [32].

Metabolic stress can trigger AMPK signalling by increasing AMP and reducing ATP levels. This pathway is activated under stressful conditions involving low energy [31], leading to an elevated cytosolic AMP-to-ATP ratio. When activated, AMPK counteracts the energy deficit by stimulating catabolic pathways that generate ATP (glycolysis and fatty acid oxidation) and suppresses anabolic pathways (triglyceride, fatty acid, transcription, cholesterol, and protein synthesis) that deplete ATP [29].

Alterations of these cellular events in diabetes and the downregulation of AMPK activity are crucial in the pathogenesis of diabetic-related complications [29]. AMPK is an upstream mediator of nuclear factor erythroid 2-related factor 2 that enhances the antioxidant defence system [31, 33]. Mitophagy AMPK/mammalian target of rapamycin pathway inhibition leads to damage of renal tubules when glucose levels are high for a short time. Therefore, activation of AMPK or mammalian target of rapamycin could halt kidney damage [34].

### Phosphatidylinositol-3-kinase (PI3K) hyperactivity

The lipid kinase PI3K plays an upstream role in the PI3K/AKT signalling pathway by modulating NF-kB. The PI3K enzyme phosphorylates protein kinase B (PKB and AKT), which regulates cell growth, proliferation, and protein synthesis [35]. AGEs activate PI3K/AKT, which enhances NF-κB and exacerbates inflammation [36]. The PI3K/Akt signalling pathway regulates DKD development. For instance, in diabetic renal tubular cells, the pathway activation regulates EMT, cell growth, and lipid metabolism [37]. DKD and many diseases show aberrant activation of the pathway [35].

Activation of this pathway may mitigate inflammation in DKD. So, alternative strategies, such as activation of the PI3K/AKT signalling pathway and subsequent inhibition of the NF-kB-mediated inflammatory response [38], may safeguard against kidney damage after diabetic onset.

## Potentiation of renal inflammation

**Fig. 2:**
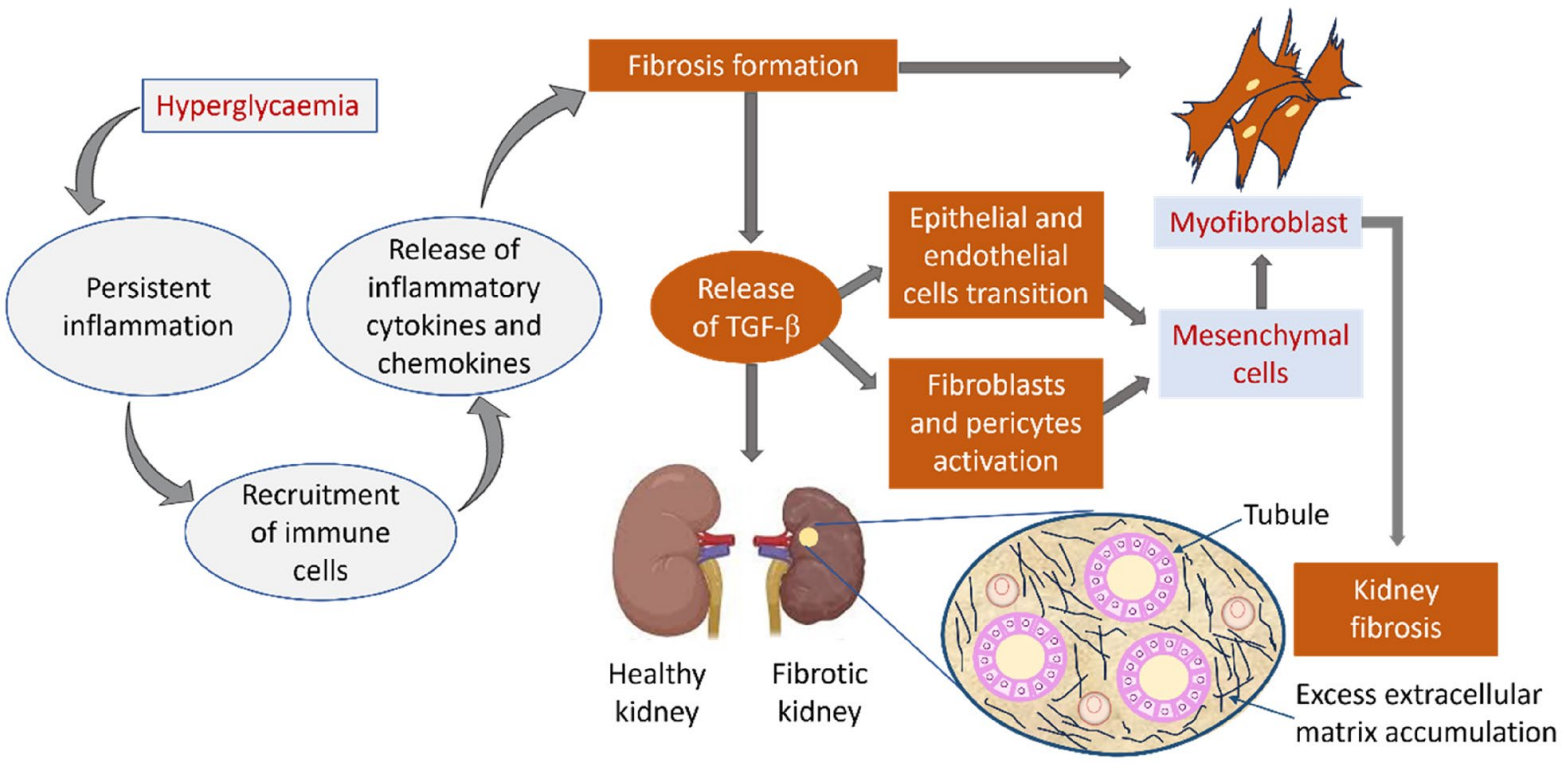
Mechanisms underpinning inflammatory processes related to fibrosis in DKD. Hyperglycaemia induces an inflammatory response in the kidneys through the recruitment of immune cells and release of inflammatory cytokines and chemokines, These mediators drive the process of fibrosis with the release of TGF-β, resulting in epithelial/endothelial cell transition and activation of fibroblasts/pericytes. This process leads to the formation of mesenchymal cells, myofibroblasts, excess extracellular matrix accumulation, and, ultimately, fibrosis of the kidney

Renal fibrosis is the primary determinant of renal pathology, and inflammation plays a pivotal role in initiating this process. Inflammation triggers the continual release of inflammatory cytokines, which triggers signal transduction events that stimulate myofibroblast activity, excess EMT deposition, and renal fibrosis, eventually leading to ESKD [39] (Fig. 2). Under inflammatory conditions, proximal tubular epithelial cells undergo phenotypic changes following EMT buildup. This results in the epithelial-mesenchymal and endothelial-mesenchymal transitions and subsequently fibroblasts and pericytes stimulation [2, 24].

### Activation of inflammation through TLRs

Inflammatory chemokines promote proinflammatory processes and leukocyte recruitment to damaged tissues. Chronic exposure to diabetes substrates causes renal cell damage in DKD. This results in cell death and the release of damage-associated molecular patterns (DAMPs), such as ROS, phosphoglyceric acid, and free fatty acid, into the extracellular space [40, 41], thereby promoting fibrogenesis, inflammation, and renal pathology [42]. TLRs and RAGE are pattern recognition receptors that detect DAMP signals [41]. TLRs recognise pathogen-associated molecular patterns (PAMPs) and DAMPs, which aid in innate immune responses against injury and infection [43].

TLRs are transmembrane proteins that convey antigen recognition information from the exterior into the cell, playing a vital role in immune responses. TLR-mediated innate immune system stimulation has a role in the pathogenesis of insulin resistance, diabetes, and atherosclerosis [40]. TLR pathway activation worsens inflammation and, as a result, accelerates the course of DKD.

TLRs represent a family of germline-encoded receptors that facilitate the development of inflammatory and immunological responses. They are expressed on diverse cell types, including antigen-presenting and kidney intrinsic cells. Among the eleven TLRs, only TLR4 is extensively expressed in intrinsic renal cells. TLR4 upregulates inflammatory kidney diseases (tubulointerstitial nephritis and glomerulonephritis), renal ischemia–reperfusion injury, and DKD [44].

The innate immune response is triggered by the recognition of TLR ligands, which in turn stimulates TLR signalling. This signal initiates M1 macrophage polarisation and infiltration, mediates NF-kB transcription, and triggers an inflammatory cascade with the release of proinflammatory cytokines and chemokines. Almost all TLRs (except TLR-3) use myeloid differentiation primary response 88 [45] as a general adapter protein when activating NF-kB. The stimulatory effects of the innate immune system on TLRs are associated with the pathophysiological process of DKD [38].

### Vasoconstriction through endothelin-1 (ET-1)

The vasoconstrictor ET-1 [46, 47] monitors vascular function. It was first identified as a downstream factor of TGF-β in a focal segmental glomerulosclerosis model, where it induced albuminuria via mitochondrial ROS in glomerular endothelial cells [48]. In experimental DKD, an elevated serum level of ET-1 was associated with increased urinary levels of N-acetyl glucosamine and albumin, which caused diabetic lesions. The impairment of glomerular endothelial mitochondria was associated with high expression of the glomerular ET-1 receptor and increased circulating ET-1 [7].

Hyperglycaemia prompts the kidneys to release nitric oxide and vascular endothelial growth factor, resulting in the dilation of the afferent glomerular arterioles and release of ET-1 and angiotensin II (Ang II). Together, ET-1 and Ang II contract the efferent arterioles, resulting in increased blood pressure and the onset of DKD [49]. In diabetes, the endothelium-dependent vasodilatation is partially decreased due to a metabolic switch that favours ET-1 signals over the vasodilating effects of nitric oxide [9]. This induces endothelial and NF-kB–mediated cytokines (IL-6 and TNF-α) to promote an inflammatory response [5]. Their overexpression disrupts endothelium-dependent regulation in diabetes, and activation of this pathway compromises endothelium function (e.g., hyperfiltration) in the renal vascular system.

The signals, pathway, mediators, and their effect on kidney failure is presented in Table 1.

## Transforming growth factor-β1 (TGF-β1) as central hub in DKD

Hyperglycaemia stimulates the transcription of TGF-β1 in different renal cells by upregulating the expression of TGF-β genes, TGF-proteins, and/or TGF-β receptors [50], hence promoting TGF-β1 production [51]. TGF-β is a multifunctional profibrogenic cytokine that essentially causes inflammation and fibrosis at high concentrations [52]. Nearly all intracellular signalling pathways associated with kidney dysfunction enhance renal TGF-β activity as an intermediary step [50]. This makes TGF-β1 one of the key mediators in the pathogenesis of DKD, a process that may be linked to a putative glucose-responsive element in the promoter region of the TGF-β1 gene.

**Fig. 3:**
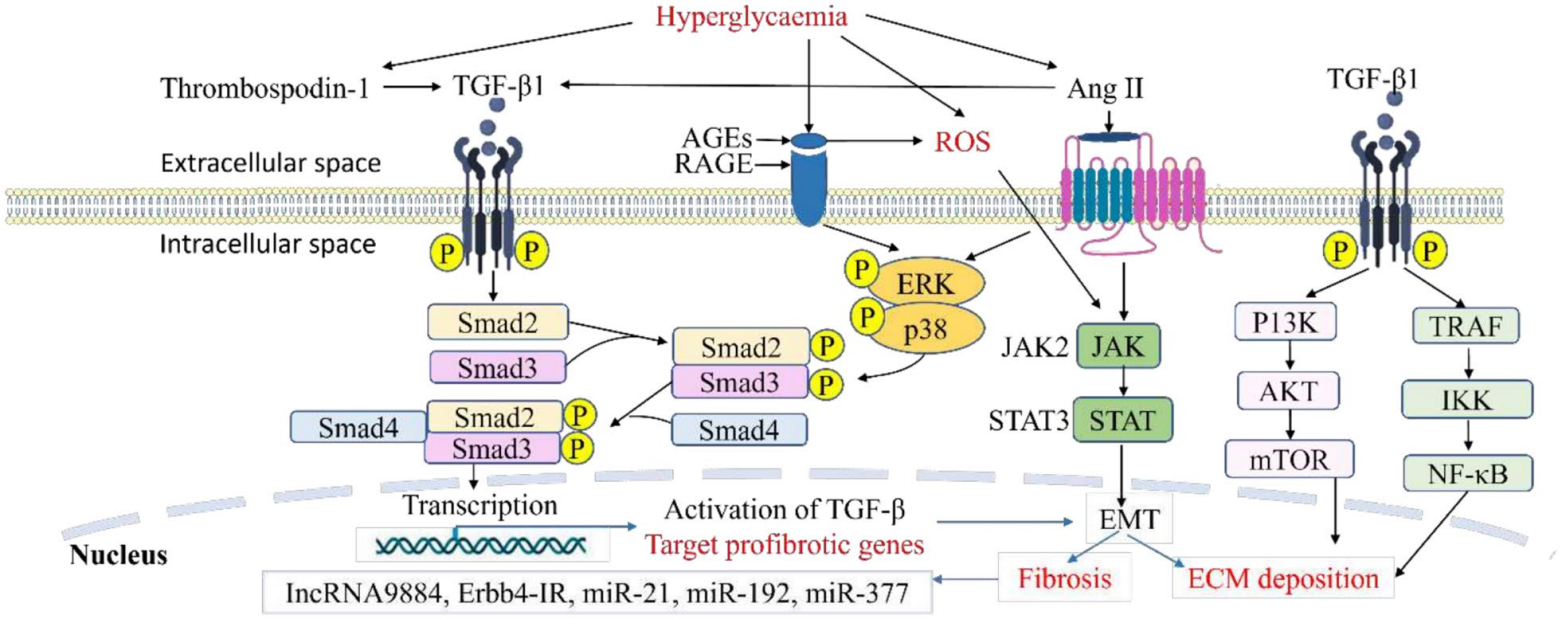
Activation of transforming growth factor 1 (TGF-β1) signalling in the development of DKD. Hyperglycaemia results in increased advanced glycation end products (AGEs) which bind to its receptor (RAGE), resulting in ROS generation in kidney cells. It also upregulates the transcription of the TGF-β1 gene, giving rise to TGF-β1 production. TGF-β1 interacts with key inflammatory and fibrotic pathways, including extracellular signal­ regulated kinase (ERK), p38, and the Smad cascade, to drive fibrotic gene transcription in the nucleus, promoting mesangial expansion and fibrosis. Hyperglycaemia further activates angiotensin II (Angil), which engages the Janus kinase-signal transducer and activator of transcription (JAK/STAT) pathway, further promoting epithelial-mesenchymal transition (EMT) formation and ultimately fibrosis. The mechanistic target of rapamycin (mTOR) and nuclear factor-kappa B (NF-κB) promotes ECM deposition as well through the non-Smad pathway

High glucose enhances TGF-β1 activity by upregulating thrombospondin 1, which activates latent TGF-βs and increases the expression of TGF-β receptor 2 (TGF-βR2) in the murine MC, independent of TGF-β-1 induction [53–55] (Fig. 3). This validates the involvement of hyperglycaemia in the stimulation of TGF-β signalling throughout the progression of DKD.

Fibrosis is an uncontrolled tissue repair process that occurs after an injury or inflammation; however, in diseases like DKD, fibrosis eventually leads to organ failure [14, 15]. Renal fibrosis is a major pathophysiological characteristic of DKD. TGF-β1 mediates renal fibrosis by promoting ECM deposition, glomerulosclerosis, and interstitial fibrosis [4, 56]. In a high-glucose milieu, TGF-β1 initiates a downstream signalling cascade that culminates in the loss of adhesion proteins and connexins, facilitating the buildup of ECM on the cell surface or intercellularly—a primary factor in the progression of renal fibrosis [57].

TGF-β1 exacerbates ECM degradation, enhances crosslinking between collagen and elastin fibres, and induces proximal tubular and endothelial cell dedifferentiation [58]. Growth differentiation factor, an inflammatory and stress-induced cytokine also known as macrophage inhibitory cytokine or placental TGF-β1, belongs to the TGF-β superfamily. Its elevated levels act as a predictive indicator of disease deterioration. Growth differentiation factor-15 exerts a suppressive effect on inflammatory responses and offers protection against DKD by curbing the activation of NF-κB, thereby making it a potential therapeutic target for nephropathy in diabetes [59]. Taken together, inhibiting TGF-β signalling is a promising potential therapeutic strategy for DKD [60].

### The crosstalk of Smad and TGF-β

TGF-β1 activates the Smad signalling pathway by phosphorylating Smad2 and Smad3. The pathway plays a crucial role in ECM accumulation and renal fibrosis development [61–63]. The product (phospho-Smad2/3) then binds to Smad4 forming hetero-oligomeric complexes [64]. These complexes translocate to the nucleus, where they bind to Smad-binding elements (SBEs) or Smad-containing complexes, modulating the transcription factors of genes encoding collagen, Smad7, fibronectin, and α-smooth muscle actin, all of which are implicated in kidney fibrosis (Fig. 3).

Furthermore, AGEs (glycated proteins, lipids, and nucleic acids) [65] induce rapid phosphorylation of Smad2 and Smad3 within 30 min through the RAGE-mediated ERK/p38-MAPK signalling crosstalk in tubular epithelial cells and MCs through a TGF-β-independent pathway (Fig. 3). AGEs enhance TGF-β synthesis, inducing the canonical TGF-β pathway that activates downstream Smad signalling in a TGF-β-dependent manner. Ang II causes deterioration of DKD by inducing the long-term activation of Smad2/3 in a TGF-β-dependent way [66, 67].

Correlation analysis reveals that renal and plasma levels of TGF-β1 are associated with the severity of renal dysfunction in patients with DKD. Because TGF-β1 is an upstream mediator of DKD via Smad signalling, inhibiting TGF-β1/Smad signalling can improve kidney function and slow the progression of DKD [53–55].

### The crosstalk of Ang II and TGF-β

**Fig. 4:**
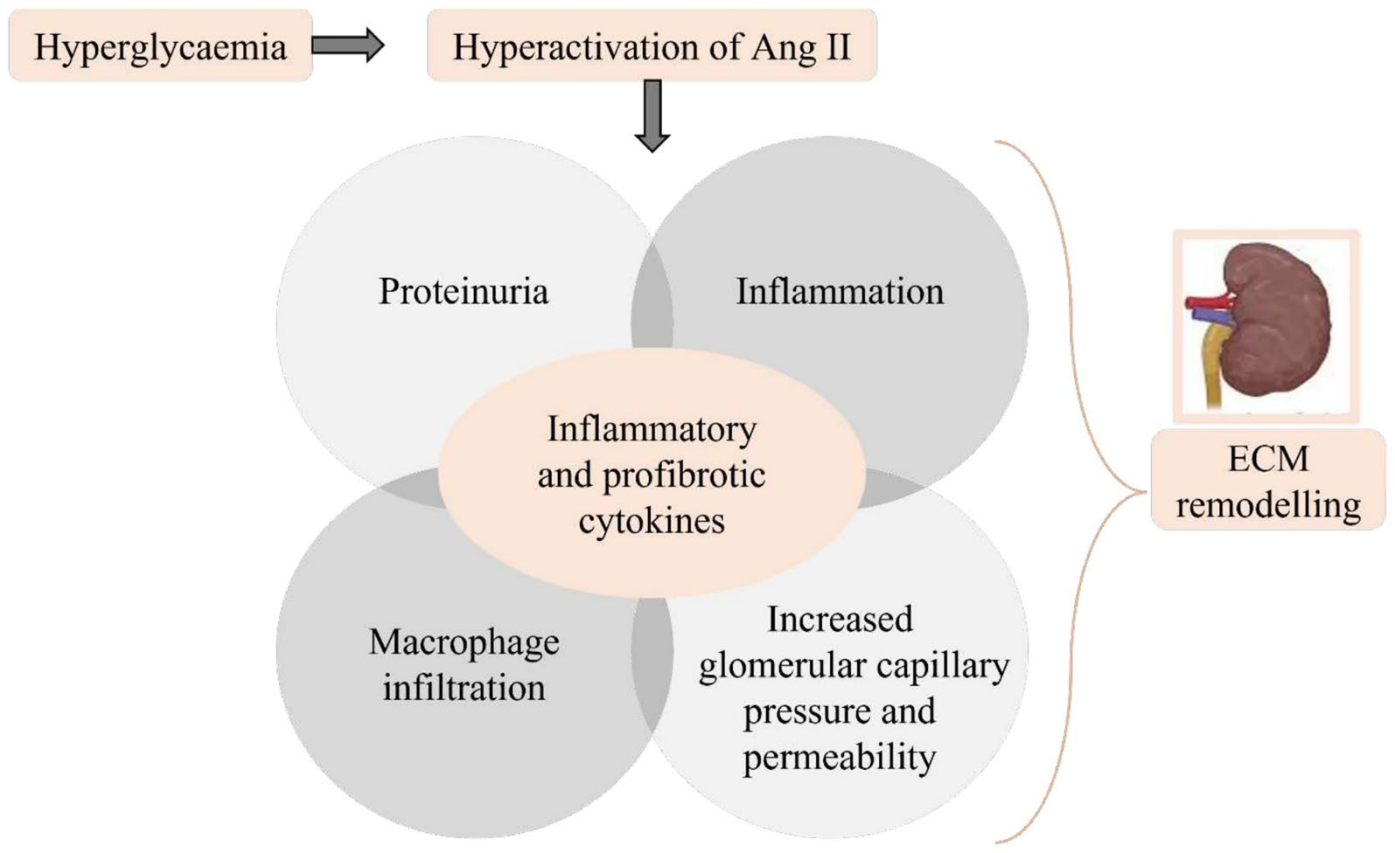
Hyperactivation of Ang II and renal extracellular matrix modelling. Chronic hyperglycaemia activates angiotensin II (Angll), which results in proteinuria, inflammation, macrophage infiltration, elevated glomerular capillary pressure and permeability. All these processes release inflammatory and profibrotic cytokines leading to extracellular matrix remodelling

Hyperglycaemia and insulin resistance increase Ang II expression causing ROS production [68] and TGF-β signalling activation [61, 62, 69] (Fig. 3). Hyperactivation of Ang II results in proteinuria, increased glomerular capillary pressure/permeability, promotion of inflammation, and macrophage infiltration. This sequence of responses culminates in the production of inflammatory and profibrotic cytokines, leading to further ECM remodelling [70] (Fig. 4).

Ang II triggers apoptosis and injury to podocytes [71] and stimulates the expression of vascular endothelial growth factor, leading to increased collagen deposition in the basement membrane [70]. It is involved in hypertension-induced fibrogenic mechanisms and is the major effector of RAAS. High Ang II levels enhances RAAS activity, creating increased mechanical stress on glomerular structures, which causes severe vascular, glomerular, and tubulointerstitial injuries by inducing hypertension and hyperfiltration along with the release of TGF-β1 through the angiotensin type 1 receptor [24].

TGF-β1 is essential for Ang II to activate fibroblasts and induce fibrosis. For individuals with CKD, blocking TGF-β1 signalling may mitigate kidney injury and enhance therapeutic efficacy [60–62]. Delaying the initiation and progression of DKD involves managing hypertension, mainly by using RAAS-blocking agents such as angiotensin-converting enzyme inhibitors or Ang II receptor blockers [72–75]. The angiotensin-converting enzyme inhibitor promotes sodium and water excretion by aldosterone inhibition and causes vasodilation of renal arterioles, whereas Ang II receptor blockers promote sodium and water excretion by binding to angiotensin receptors [76].

### The crosstalk of JAK/STAT and TGF-β

The JAK/STAT signal transducer pathway is another vital signalling cascade involved in the development of DKD through the TGF-β up- and downstream signals [14, 15, 80, 77–79]. JAK/STAT signalling upregulates TGF-β expression and Smad-independent TGF-β pathways, hence exacerbating fibrosis (Fig. 3). The pathway is exaggerated when TGF-β triggers cytokines like IL-6, which further activate the JAK/STAT pathway, amplifying inflammation and fibrosis [14, 15]. The pathway consists of four JAK and seven STAT family members. It is an essential intracellular signalling pathway of cytokines and other stimulants that regulate gene expression, cell activation, proliferation, differentiation, EMT, and fibrosis in DKD. Early stages of DKD are characterised by an increase in JAK messenger ribonucleic acid (mRNA) transcripts, while advanced stages exhibit downregulation [38]. The activation of JAK/STAT signalling facilitates Ang II-mediated MC proliferation and enhances TGF-β production, which worsens excessive ECM secretion and aggravates the pathophysiology of DKD [81].

The JAK/STAT pathway is important in different renal cell types, where it transduces diverse signals from extracellular ligands, including cytokines, chemokines, growth factors, and hormones [74]. The JAK/STAT pathway is a key contributor to the initiation and advancement of DKD, promoting the excessive proliferation and development of glomerular MCs, which ultimately results in renal failure in diabetes [57, 77, 82].

The JAK/STAT cascade is an intracellular signalling mechanism associated with cytokines, serving as a crucial mediator between paracrine signals and nuclear receptors. The mechanism is activated by cytokines and diabetic factors relevant to DKD pathogenesis. The upregulation of JAK/STAT occurs in the glomerular cells of patients with early DKD. The tubulointerstitial expression of various JAK and STAT isoforms increases with disease progression and exhibits an inverse correlation with the estimated GFR [83]. Increasing the activity of phosphorylated STAT3 or STAT3 can promote the proliferation of renal interstitial fibroblasts and advancement of renal fibrosis [82].

The stimulation of the JAK/STAT pathway is a significant mechanism by which hyperglycaemia induces kidney injury. For instance, JAK/STAT signalling in glomerular MCs promotes excessive renal cell proliferation and enhances the synthesis of TGF-β1, collagen IV, and fibronectin, all of which contribute to glomerulosclerosis in DKD [14, 15, 84, 85]. Gene and protein expression studies of kidney biopsies obtained from patients diagnosed with early or advanced stages of the disease have shown increased activation and expression of JAK/STAT. Abnormal JAK/STAT signal functions as an upstream regulator of TGF-β1 signalling [58].

Elevated ROS levels resulting from hyperglycaemia activate JAK2, resulting in an increased expression of TGF-β1. Additional stimuli that activate TGF-β1 include mechanical stretch, AGEs, and thrombospondin-1 [60, 64, 86]. Additionally, interleukin-like kinase, Smad2/3 complex, PKC, p38-MAPK, and Wnt/beta-catenin signalling are among the downstream targets that mediate profibrogenic effects of TGF-β1 [58].

Gene expression and activity of JAK1 and JAK2 are linked with the advancement of CKD in diabetes. In patients with DKD, increased mRNA expression of various JAK/STAT components in the glomerular and tubulointerstitial compartments was adversely linked with the estimated GFR [74]. The negative modulation of JAK/STAT can inhibit hyperglycaemia-induced renal damage, consequently enhancing renal function, decreasing renal inflammation and fibrotic lesions, and reducing the progression of DKD [14, 15, 77].

Both inhibitors of JAK (baricitinib, tofacitinib, and ruxolitinib) [11, 14, 15] and TGF-β (neutralizing anti-TGF-β1 antibodies such as fresolimumab and IgG4κ monoclonal antibody and TGF-β receptor kinase inhibitors like galunisertib and vactosertib) mitigate inflammation in DKD [87–89]. The concurrent inhibition of JAK/STAT and TGF-β/Smad may therefore yield synergistic advantages in mitigating fibrosis and inflammation in DKD.

### The crosstalk of NF-κB and TGF-β

The NF-κB pathway is an important mechanism implicated in the pathology of the kidneys [74]. It is the principal transcription factor essential for inflammatory processes in the diabetic kidney [36, 90, 91], and is the first step towards promoting TGF-β1 transcription. In diabetic state, TNF-α and IL-6 enhance IκBα phosphorylation, which is then degraded by the proteasome to release p65/p50 (NF-κB heterodimers) into the nucleus for TGF-β1 transcription and amplified stimulation of TGF-β1 expression [92]. NF-κB itself and the inflammatory cytokines it induces (e.g., IL-1β, TNF-α, and monocyte chemoattractant protein-1) can further enhance TGF-β synthesis and signalling, upregulating TGF-β expression and establishing a positive feedback loop that sustains fibrosis and inflammation. The activation of NF-κB in kidney cells induces the production of TNF-α and IL-1β, which in turn enhances NF-κB through the positive feedback loop. NF-κB then translocate into the nucleus, to promote genes transcription involved in immune response, inflammation, and fibrosis [39, 53–55, 93].

NF-κB tightly interacts with inhibitory proteins, IκB and IκB kinase, which are upstream modulatory elements in the transduction cascade of NF-κB signals. Upon activation of NF-κB by upstream signals such as hyperglycaemia, AGEs, inflammatory cytokines, albuminuria/proteinuria, Ang II, OS, and mechanical stress [74, 83], it dissociates from its inhibitor IκB proteins and is translocated into the nucleus [36]. This promotes the transcription of proinflammatory factors, including TNF-α, IL-1β, IL-6, and monocyte chemoattractant protein-1 [38, 83]. IL-6 causes GBM thickening, growth, proliferation, and activity of MC, ECM accumulation, and glomerulosclerosis. TNF-α promotes sodium reabsorption through the activation of epithelial sodium channel in renal distal tubule, stimulate release of TGF-β, and causes renal hypertrophy. It also causes cell death via autocrine and direct mechanisms, which alters permeability of the renal endothelial cells. TNF-α increases albumin permeability by causing dysfunction of the glomerular capillary wall barrier function [94]. Monocyte chemoattractant protein-9 enhance EMT and the deposition of ECM through direct activation of myofibroblasts [95].

NF-κB activation induces the expression of additional target genes, namely inducible nitric oxide synthase and intercellular adhesion molecule 1. These genes enhance inflammation, induce excessive fibronectin synthesis, and facilitate ECM accumulation, ultimately advancing the progression of DKD [32]. Elevated expression of receptor activator of NF-κB (RANK) in the podocytes of individuals with DKD contributes to podocyte damage. The mucin domain-3 and T cell immunoglobulin domain are also involved in processes that damage the podocytes, thus acting as essential regulators of inflammatory processes in DKD [96].

### Connective tissue growth factor (CTGF), TGF-β1, and cell death

Connective tissue growth factor (CTGF) also known as CCN2 is a downstream component in DKD pathophysiology associated with structural renal transitions in the early and late phases of the condition [18]. It is a profibrotic cytokine secreted by renal cells in response to hyperglycaemia. CTGF facilitates extracellular matrix synthesis, cellular migration, and interstitial matrix deposition via EMT in people with diabetes [12, 97].

Activation of Smad2/3 raises the expression of certain profibrotic genes, such as CTGF, which are targets of TGF-β and promote the shift from autophagy to senescence [89]. Elevated renal tubular epithelial cell senescence caused by hyperglycaemia is a significant biological occurrence preceding renal interstitial damage in DKD [98, 99]. Cellular senescence and senescence-associated secretory phenotype are involved in the pathogenesis of CKD. CKD in turn facilitates the progression of cell senescence and the secretion of senescence-associated secretory phenotype. The p16 protein (cell cycle inhibitor) and senescence-associated β-galactosidase were observed to be highly upregulated in the glomeruli, interstitium, and tubules of various kidney diseases including DKD. The participation of senescence markers in diabetic kidney tissues was confirmed in different experimental models [100].

CTGF production can also be stimulated by increased concentrations of AGEs in renal cells in diabetes [101]. CTGF, as a downstream effector of TGF-β, interacts with TGF-β to promote fibrosis in DKD [60].

The CTGF gene plays an important role in cell proliferation and is a direct target of Yes-associated protein (YAP). It regulates cell growth, proliferation, and apoptosis, hence playing an essential function in tissue regeneration, organ enlargement, tumorigenesis, and cancer development. Research findings observed upregulation of CTGF, TEA domain (TEAD), and YAP in glomerular cell nuclei of patients with DKD [102]. An increased TGF-β/CTGF signalling correlates with DKD advancement via the downregulation of miR-26a [103].

## Cellular death sentence characterised by apoptosis and autophagy dysregulation

### Hippo signalling, the dual-edged sword pathway

**Fig. 5:**
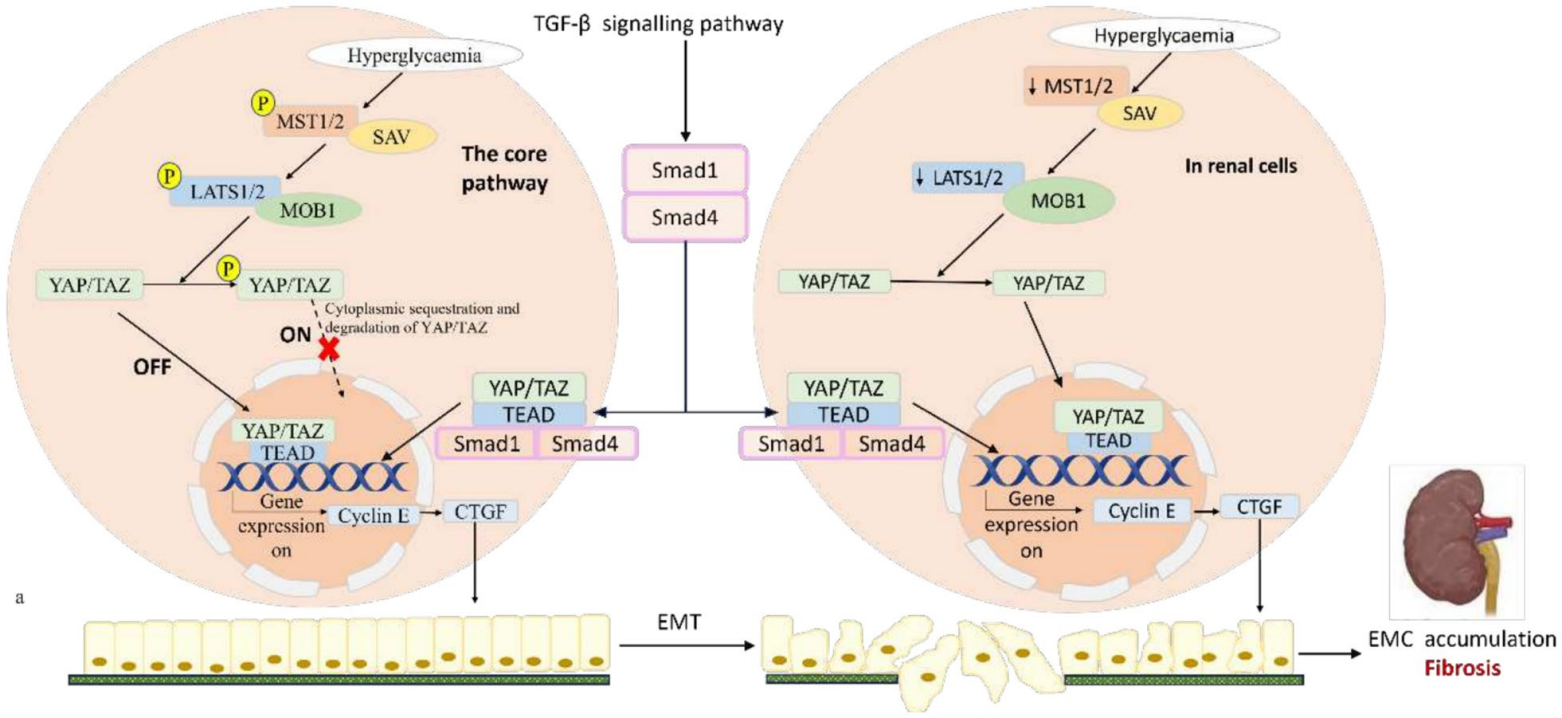
Hippo signalling contributes to renal fibrosis and damage in diabetic conditions. The core Hippo pathway comprises a kinase cascade involving MSTl/2 and LATSl/2. In diabetic conditions, when the pathway is switched on, YAP/TAZ phosphorylate, leading to YAP/TAZ inhibition, thus preventing their nuclear translocation and downstream gene activation. When the pathway is off, YAP/TAZ translocates to the nucleus to activate downstream genes involved in fibrosis. Dysregulated Hippo signalling in the diabetic kidney results in decreased MSTl/2 activity, which favours YAP/TAZ translocation to the nucleus, where they promote fibrosis by activating profibrotic genes such as CTGF and TGF-β1. The TGF-β signalling pathway via Smad 1 and 4 (Smad-dependent and non-Smad pathways) activates the translocation of the Smad 1/4/YAP/TAZ complex into the kidney, which upregulates fibrotic markers, leading to epithelial-mesenchymal transition (EMT) and extracellular matrix (ECMJ accumulation, thereby amplifying renal fibrosis

The Hippo pathway is a key regulator of apoptosis, fibrosis, and inflammation in DKD. Dysregulation of its components, especially MST1/2 and YAP/TAZ, contributes to renal cell injury and progressive kidney damage. The Hippo pathway is an evolutionarily highly conserved protein kinase cascade [102, 104]. It consists of three interdependent modules: an upstream regulatory module (KIBRA and merlin/NF2) [105, 106], a core kinase module [mammalian Ste20-like serine/threonine kinases 1/2 (MST1/2) and large tumour suppressor 1/2 serine/threonine protein kinases (LATS1/2)], and a downstream transcription module [Salvador homology (SAV) and monopolar spindle-one-binder protein– Mps-one binder 1 (MOB1)]. The activation of the pathway results in LATS1-mediated phosphorylation of the transcriptional coactivators YAP and transcriptional co-activator with PDZ-binding motif (TAZ), which are the downstream effectors. This culminates in the inactivation of YAP/TAZ by proteasome-mediated degradation and/or cytoplasmic sequestration. On the other hand, Hippo pathway inhibition leads to decreased serine phosphorylation of MST1 and LATS1, activates YAP, resulting in the nuclear translocation and accumulation of YAP/TAZ. This facilitates downstream target gene expression as a transcriptional co-activator via interaction with transcription factors, such as members of the TEAD family. This favours the expression of target genes such as cell cycle protein E (cyclin E) [35, 107], epithelial-interstitial induced transformation of renal tubular epithelial cells, which in turn, overproduce and secrete large quantities of ECM, including type IV collagen and laminin. These processes ultimately result in renal fibrosis, nephron loss, and chronic kidney failure [107, 108] (Fig. 5).

The transcriptional regulators YAP and TAZ, which are also called WW Domain-Containing Transcription Regulator 1 (WWTR1), are the primary effectors of the pathway [108]. They modulate cell growth, proliferation, and apoptosis by controlling the expression of downstream genes such as cell cyclin E [20, 102], which highly influence organ size, tissue regeneration, embryo development, and tumour development [35, 104]. Hippo upstream MST1 kinase is a pivotal regulator of β-cell death and dysfunction in diabetes [109]. It is also a key mediator in fibrosis development and growth of fibrosis in tissues [107].

Hippo pathway is linked to renal fibrosis, DKD, and other kidney diseases [102]. Renal fibrosis is the common pathway that culminates in ESKD and is a major biomarker of renal insufficiency. In diabetic conditions and DKD, when there is alteration in the biomechanical properties of tissues, the YAP/TAZ sensor gets activated, causing the release of proinflammatory and profibrogenic signals, further exacerbating renal inflammation [35].

Under diabetogenic conditions, significant overexpression and autophosphorylation of MST1 in response to various chronic diabetes stimuli have been demonstrated in vitro and in vivo. Complete restoration of β-cell viability following the suppression of MST1 activation was achieved by the triple kinase (Her2/EGFR/MST1) inhibitor neratinib [110]. A review reported that pre-diabetes and diabetes stimulated MST1, resulting in its significant autophosphorylation, activation of programmed cell death, and apoptosis [111].

Conversely, glucose induced MST1 inactivation in renal tubular cells, results in YAP activation, its translocation into the nucleus, and EMT of renal tubular epithelial cells, resulting in renal fibrosis and chronic renal failure [107] (Fig. 5). In vitro findings demonstrated inactivation of the Hippo pathway in MCs cultured in high glucose, leading to increased proliferation of glomerular MCs. The study revealed that reduced phosphorylation of MST1 and LATS1 enhanced the feedback loop, subsequently increasing the expression of downstream genes such as cyclin E [104]. Additional studies showed increased MC proliferation following decreased phosphorylation of MST1 and LATS1 and increased PI3K/Akt activation in diabetic mice and high glucose-treated MCs [35]. Human MCs exposed to high glucose milieu exhibited reduced phosphorylation of LATS1, which correlated with diminished phosphorylation of its target YAP [112].

In induced acute kidney injury, the inactivation of SAV1 in renal tubule cells leads to the progression of renal interstitial fibrosis. Deletion of SAV1 in tubular epithelial cells specifically enhanced the presence of myofibroblastic EMT-like cells and exacerbated tubulointerstitial fibrosis [108]. Inhibition of Hippo signalling in glomerular MCs occurs during the initial phases of nephropathy in diabetes; with enhanced proliferation of glomerular MCs and accumulation of ECM in diabetic rats [20]. MST1 downregulation occurred in a glucose- and time-dependent manner with the attendant metabolic consequences in type 1 and 2 diabetes [102]. This may point to the acute and chronic expression of Hippo signal in DKD. Persistent hyperglycaemia induced inhibition of the Hippo kinases in the kidneys and enabled YAP/TAZ translocation into the nucleus, resulting eventually in fibrosis, a characteristic feature of DKD [113]. Tubule-specific MST1/2 double knockout exacerbated CKD progression by activating the inflammatory cascade [106].

The Hippo transduction pathway is a notable druggable target for managing DKD. For instance, studies show that the YAP inhibitor verteporfin inhibits YAP-TEAD interaction, consequently reducing kidney fibrosis [113]. Inhibition of SGLT2 reduced excessive glucose reabsorption and diminished the persistent hyperactivation of YAP/TAZ elicited by a high glucose environment. Dapagliflozin, a therapeutic agent, facilitates YAP/TAZ phosphorylation, leading to their cytoplasmic retention, deactivation, and destruction [5, 114]. YAP/TAZ possess regenerative functions, so complete inhibition may have unintended consequences.

While these findings from different experimental models align with the established role of the Hippo pathway in fibrotic processes, we see a Hippo pathway-unique kidney-specific response to chronic hyperglycaemia that differs from the β-cell. However, there exists a significant gap in the understanding of how acute versus chronic hyperglycaemia differentially impacts this pathway in the kidney. Addressing this gap, especially for novel therapeutic strategies in diabetes, will be of great importance.

### Notch signalling

Notch signalling is associated with the progression of DKD [14, 15, 60, 63, 82]. Notch is a transmembrane receptor consisting of an intracellular domain known as the Notch intracellular domain and an extracellular domain. A γ-secretase cleaves the Notch intracellular domain to promote free NICD translocation into the nucleus [115, 116]. The transduction signal system in mammals comprises four transmembrane receptors “(Notch 1, 2, 3, and 4), three delta-like ligands (DLL1, DLL3, and DLL4), and two Jagged family ligands (JAG1 and JAG2). Under pathological conditions, the Notch pathway is activated, influencing processes such as apoptosis, cellular proliferation, and EMT. When the ligand of Notch pathway binds to its receptor, Notch gets activated to form the Notch intracellular domain. This translocates to the nucleus to regulate downstream targets expression and ECM/EMT induction. This ultimately results in renal fibrogenesis in DKD [57].

The Notch signalling pathway participates in fibrosis mechanisms in many organs. Increased Notch 1, Jagged-1, and Notch 3 expression has been observed in pulmonary fibrosis, accompanied by increased mesenchymal markers and decreased epithelial biomarkers. Hypoxia-induced EMT in renal tubular epithelial cells facilitates the direct targeting of Notch 1 and Jagged-1, along with the subsequent activation of Notch downstream signal [115, 116]. TGF-β induces Notch 1, which then activates p53 and Cdk1a and drives cell death and glomerulosclerosis. The Notch 1-induced podocyte cell death entails the transition of podocytes from a dormant state to cell-cycle re-entry upon stimulation by growth hormone or TGF-β [9]. Snail, which is one of the downstream genes of Notch signalling, plays a critical role in fibrosis induction. It serves as a link for EMT induction in renal tubular epithelial cells and activates the pathway. Upregulation of the Snail promoter is activated by the Notch pathway, and its high expression has an inverse relationship with E-cadherin expression with increased α-Smooth Muscle Actin synthesis [57].

### Wnt/β-catenin pathway activation

The Wnt signalling cascade is categorised into canonical (β-catenin-dependent) and noncanonical (β-catenin-independent) pathways [117]. β-catenin functions as the primary intracellular mediator of canonical Wnt signalling and acts as a principal transcriptional regulator controlling the expression of all RAAS genes in diseased kidneys [118]. Appropriate β-catenin expression is important for sustaining the glomerular filtration barrier and its functionality [119]. The Wnt/β-catenin signal transduction pathway plays a crucial role in organ development, tissue homeostasis, and injury repair of multicellular organisms [42, 120].

The Wnt/b-catenin cascade is silent in normal adults but is activated after kidney injury [16, 120–122]. This pathway contributes to the initiation and progression of chronic renal impairment by activating the expression of downstream cytokines that induce renal interstitial fibrosis [123]. The pathway is intricately connected to the formation of tubulointerstitial fibrosis by transdifferentiation of renal tubular epithelial cells in DKD. Hyperglycaemia activates the pathway in renal tubular epithelial cells and upregulates the expression of the related proteins, which further increases renal tubulointerstitial fibrosis and causes renal injury in patients with diabetes [124]. The activation of Wnt/β-catenin signals exacerbate podocyte failure in DKD [119].

### MicroRNA dysregulation

MicroRNAs (miRNAs) are a class of endogenous, single-stranded, approximately 22-nucleotide, noncoding RNA molecules that function as developmental regulators [13, 115, 116, 125]. They inhibit the expression of target genes through incorrect base pairing with the 3'-untranslated regions of target mRNAs, resulting in translational repression and/or mRNA degradation [126]. They regulate physiological and pathological events by post transcriptionally suppressing gene expression [127, 128] by obstructing translation or enabling the cleavage of specific target mRNAs or transcriptionally, via targeting of the promoter region [125]. They participate in proliferation, differentiation, apoptosis, development [129], immunity, metabolism, oncogenesis, and viral replication [130].

Many miRNAs are expressed in different parts of the kidney, where they regulate multiple functions essential for sustaining normal renal physiology [131]. Numerous miRNAs are implicated in the tissues associated with diabetic complications, with the kidney possessing a higher concentration relative to other organs [125, 126, 131, 132]. For instance, many miRNAs are involved in critical roles in the onset and progression of diabetes [103] and DKD [8, 125, 127] by participating in insulin resistance, inflammation, fibrosis, hypertrophy, endoplasmic reticulum stress, autophagy, OS, and podocyte injury [133].

Five miRNAs (miR-192, miR-194, miR-204, miR-215, and miR-216a) are identified to be enriched in the kidney relative to other organs. Additional kidney-specific miRNAs include miR-146a and miR-886 [131]. This indicates their potential function in the kidney [126]. Other miRNAs found in the kidney and other organs are miR-21, miR-200a, miR-196a/b, miR-10a/b, miR-30a-e, miR-872, miR-130, miR-143, and let-7a-g [131]. Many more noncoding RNAs are participants in DKD development [115, 116, 128, 132, 134]. miRNAs provide a regulatory function in signal transduction associated with DKD pathology [132]. For example, miR‐192 binds to zinc finger E-box binding homebox1/2 and activates the TGF‐β signalling pathway, resulting in renal fibrosis and proteinuria. When miR‐21 binds to phosphatase and tensin homolog, the AKT signalling pathway becomes overactivated, leading to renal hypertrophy and fibrosis. The overexpression of these nephropathy‐inducing miRNAs was observed in diabetic kidneys susceptible to DKD. For instance, miR‐181b was found to be substantially upregulated in DKD [129, 135].

TGF-β increases miRNA-21 through the Smad3-dependent cascade. miRNA-21 in turn induces renal damage by targeting Smad7. During the initial phase of DKD, dysregulated miRNAs mostly promote the expression of ECM proteins, whereas in the later phase, apoptosis and necrosis of tubular cells are evident [13]. The TGF-β/Smad3 signalling pathway regulates several miRNAs and lncRNAs to mediate DKD. Furthermore, miRNA-196b-5p present in extracellular vesicles enhances fibroblast proliferation and upregulates many fibrotic factors [14, 15].

Various miRNAs bind directly to the 3′ untranslated region of the SMAD7 mRNA, causing decreased protein levels and promoting high TGF-β signalling activity. This leads to downregulation of SMAD7 protein thus, potentiating TGF-β pathway resulting from reduced negative feedback from SMAD7. Additionally, certain miRNAs target and downregulate PTEN and SMAD7 involved in fibrosis development [136].

### Intrarenal hypoxia

Endogenous ligands are significantly upregulated in hyperglycaemia, hypoxia, and hyperlipidaemia, which are pivotal to the pathophysiology of DKD [44]. Diabetes and DKD induce several metabolic and haemodynamic stressors, such as hypoxia and hyperfiltration [33, 45]. Regardless of the aetiology of nephropathy, irreversible kidney injury progresses as CKD advances, with the vicious cycle of tubular interstitial hypoxia recognised as the final common conduit for this advancement [71].

Proximal tubule cells exposed to high glucose concentrations undergo increased apoptosis after ATP depletion or severe hypoxia [137]. Kidney hypoxia contributes significantly to the advancement of DKD. In individuals with diabetes, hyperglycaemia increases the energy requirements of tubular cells due to glomerular hyperfiltration and the upregulation of sodium–glucose cotransport. The loss of peritubular capillaries and interstitial fibrosis impairs oxygen delivery, resulting in an imbalance between oxygen demand and supply [70]. Hypoxia is a critical microenvironmental factor in the development of tissue fibrosis. Under prolonged high glucose load of DKD, the oxygen consumption of kidney tissue rises, resulting in the formation of renal interstitial fibres due to chronic hypoxia, mostly mediated by HIF-1α [53–55].

Hypoxia induces EMT through HIF-α accumulation [115, 116]. HIF-1α functions during normal development and in pathological conditions linked to reduced oxygen availability. The kidney is susceptible to hypoxic injury due to an arteriovenous oxygen shunt. Hyperglycaemia elevates mitochondrial oxygen consumption, leading to cellular hypoxia and the production of ROS [138].

HIF-1α is a key transcription factor in the hypoxic response [71] and a regulator of cellular oxygen homeostasis, which is aberrantly expressed in the serum of people with diabetes and kidneys of patients with DKD. HIF-α is strongly associated with the progression of interstitial renal fibrosis. It also regulates the expression of heme oxygenase-1, its downstream target [139].

The latent mechanism for hypoxia-induced EMT entails the inactivation of prolyl hydroxylases, resulting in the accumulation and activation of HIF-α, which subsequently promotes EMT-related gene expression such as Snail1, Twist1, and Bmi1. Upon hypoxia stimulation, HIF-α activates the TGF-β/Smad and PI3K/Akt pathways, inducing renal and pulmonary EMT, respectively. The expression of Bmi1 directly and indirectly promotes Twist1 expression, subsequently stabilizing the E-cadherin repressor Snail1 [115, 116]. Altering oxygen levels and activating hypoxia signalling via HIF-α may serve as a significant initiator and regulator of EMT [138].

## Conclusion

Globally, DKD is a significant healthcare condition with an enormous economic burden, as its prevalence rises in tandem with the incidence of diabetes. Many integrated signalling pathways modulate the metabolic perturbations responsible for the disease's pathogenesis. In this review, we elucidate how hyperglycaemia-induced metabolic perturbation kicks off molecular dysfunction that results in inflammation. The review shows how molecular mediators and intracellular signals resulting from inflammation interact synergistically, contributing to irreversible pathophysiological alterations in the kidney. These changes include the formation of myofibroblasts, different collagen types, ECM buildup, and, ultimately, tubulointerstitial fibrosis. The role of TGF-β as an upstream and downstream mediator in the progression of DKD is highlighted. Also shown is the kidney-specific Hippo pathway response to hyperglycaemia, which could give more insight for further research and therapeutic intervention. The review underscores the pathways that underlie the progression of DKD. Understanding of this mechanistic insight could enable and improve therapeutic interventions in managing the disease. While these pathways are recognised as potential drug targets for the disease, translating these findings into effective clinical interventions remains challenging. Future research should explore more targeted therapies that modulate these pathways and mediators in a multifaceted manner with minimal off-target effects.

This review is limited because other kidney diseases were not considered. Furthermore, the review did not explore the comparative effect of acute and chronic kidney failure on the signal transduction pathways. Additionally, there is a need to explore if recent therapeutic interventions are capable of completely reversing hyperglycaemia-induced kidney failure.

## Data Availability

No datasets were generated or analysed during the current study.

## Abbreviations

AGEs: Advanced glycation end-products
AKT: Protein kinase B
AMP: Adenosine monophosphate
AMPK: AMP-activated protein kinase
Ang II: Angiotensin II
cAMP: Cyclic adenosine monophosphate
CKD: Chronic kidney disease
DAMPs: Damage-associated molecular patterns
DLL1, DLL3, DLL4: Delta-like ligands
DKD: Diabetic kidney disease
ECM: Extracellular matrix
EMT: Epithelial-mesenchymal transition
ESKD: End-stage kidney disease
ET-1: Endothelin-1
ERK: Extracellular signal-regulated kinase
GFAT: Glutamine: fructose-6-phosphate-amidotransferase
GFR: Glomerular filtration rate
HIF-1α: Hypoxia-inducible factor-1α
IL: Interleukin
JAK-2: Janus kinase 2
JAG1 and JAG2: Jagged family ligands
MC: Mesangial cells
MOB1: Monopolar spindle-one-binder protein 1
MST1/2: Mammalian Ste20-like serine/threonine kinases 1/2
mRNA: Messenger ribonucleic acid
NADPH: Nicotinamide adenine dinucleotide phosphate hydrogenase
NF-kB: Nuclear Factor-kappa B
OS: Oxidative Stress
PI3K: Phosphoinositide 3-Kinase
PI3K/Akt: Phosphoinositide 3-Kinase/Protein Kinase B
p38-MAPK: P38 mitogen-activated protein kinase
PAMPs: Pathogen-associated molecular patterns
PKB: Protein Kinase B
PKC: Protein Kinase C
RAAS: Renin–angiotensin–aldosterone system
RAGE: Receptor for Advanced Glycation End-products
RANK: Receptor activator of NF-κB
ROS: Reactive oxygen species
Smad: Suppressor of Mothers Against Decapentaplegic
STAT3: Signal transducer and activator of transcription 3
TAZ: Transcriptional coactivator with PDZ binding motif
TEAD: TEA domain
TGF-β: Transforming growth factor-beta
TLRs: Toll-like receptors
TNF- α: Tumor necrosis factor alpha
YAP: Yes-associated protein
